# Intermittent BRAF Inhibition Can Achieve Prolonged Disease Control in BRAF Mutant Melanoma

**DOI:** 10.7759/cureus.410

**Published:** 2015-12-16

**Authors:** Tania Jain, Alan Bryce

**Affiliations:** 1 Internal Medicine, Mayo Clinic, Scottsdale, AZ; 2 Hematology Oncology, Mayo Clinic, Scottsdale, AZ

**Keywords:** melanoma, braf inhibitor

## Abstract

BRAF V600E is the most common somatic mutation seen in patients with metastatic melanoma. BRAF inhibitors (BRAFi), along with MEK inhibitors (MEKi), have been shown to improve overall survival in these patients with a median time to resistance of 6-10 months. We describe a patient with an ongoing response of 48 months on intermittent BRAFi therapy. She was started on vemurafenib at initial diagnosis, which was discontinued after a total of 39 weeks of therapy, and achieved a complete response due to cumulative toxicity. Upon evidence of progression on serial imaging following 81 weeks of disease-free status, BRAFi was resumed with dabrafenib, along with trametinib. Complete response was seen with seven weeks of treatment. Therapy was discontinued again, due to side effects, with an intention to pursue intermittent therapy. Serial imaging, so far, has shown no progression or recurrence of disease after over a year (66 weeks and ongoing) since discontinuation of therapy. This case underscores the clinical feasibility of intermittent BRAFi therapy in patients while still achieving a prolonged response. Disease control of 48 months, to date, has been achieved using therapy only “as needed” and keeping toxicities to the minimum.

## Introduction

The incidence of melanoma has continued to increase in the past two decades. Melanoma is a leading cause of death from cutaneous malignancies in the United States. The most common somatic mutation seen in melanoma is the BRAF V600E kinase mutation, seen in around 50% of melanoma patients. BRAF inhibitors (BRAFi) have shown significant tumor response and improved overall survival (OS) in these patients [1], notwithstanding the chronic toxicities and drug resistance despite initial response [2]. BRAFi monotherapy yields a median progression-free survival (PFS) of around six months while combined BRAFi and MEK inhibitors (MEKi) yields a median PFS of approximately 10 months [1, 3]. The median OS in this population is only 14-18 months with a three year OS on the order of 26% [4]. We describe a patient with a metastatic melanoma who has enjoyed an ongoing response to BRAFi for a total of four years and counting. This extreme response is all the more remarkable given that she has been treated with intermittent BRAFi. When considering dose reductions, the patient's total exposure to BRAFi amounted to only 19 dose-weeks of therapy {calculated as full dose x 1 week; 50% dose x 7 weeks; 25% dose x 31 weeks, and full-dose dabrafenib (D) + trametinib (T) x 7 wks = 19.25 weeks} or 9% dose drug exposure over time (weeks of drug x % of total dose/duration of ongoing management with BRAFi) to achieve this response.

Informed patient consent was obtained for treatment and also for publication of the case.

## Case presentation

A 67-year-old woman was evaluated for a left flank mass and axillary swelling. Excisional biopsy of the mass and lymph node dissection revealed metastatic melanoma with the involvement of all four axillary lymph nodes. A left periorbital lesion previously diagnosed and treated as squamous cell carcinoma at an outside hospital with resection and subsequent radiation was also retrospectively read as metastatic melanoma by our institution’s pathologist. A subsequent PET scan demonstrated areas of fluorodeoxyglucose (FDG) uptake in cervical, mediastinal, and inguinal lymph nodes, as well as in the right tibia and multiple subcutaneous nodules. BRAF analysis demonstrated a V600E mutation, and thus, she was started on vemurafenib at 960 mg twice a day. After a week of treatment, she developed cutaneous toxicity manifested by erythema noted diffusely and folliculitis on her head, trunk, and extremities, along with blistering on the soles of her feet. The dose was reduced by 50% while she was treated with prednisone for the cutaneous symptoms. However, upon taper of steroids, her skin manifestations returned with a limitation of ambulation secondary to hyperkeratotic lesions on feet, so the dose was further reduced by 25% at week 8.

**Figure 1:**
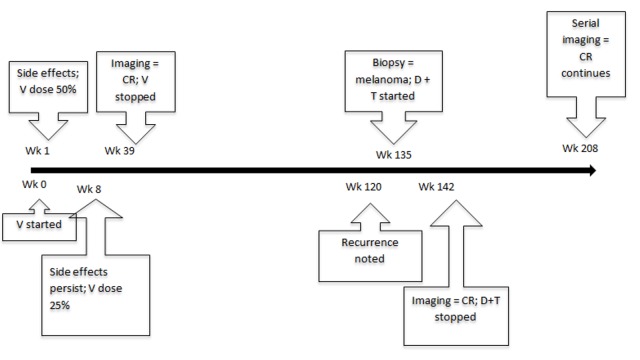
Timeline of events Wk = week; V = Vemurafenib; CR = Complete remission; D = Dabrafenib; T = Trametinib

After a total of nine months of treatment, whole body PET/CT and MRI scan of the head demonstrated a complete response and the vemurafenib was discontinued due to cumulative toxicity. The cutaneous toxicity gradually improved off therapy, although with the persistence of burning mouth syndrome. Serial imaging continued to demonstrate disease stability for 19 months. The disease then recurred with subcutaneous trunk nodules and lymph nodes in the pelvis. Biopsy confirmed the recurrence of melanoma. Since combined BRAFi and MEKi had been FDA approved in the interim, the patient was started on dabrafenib and trametinib with the intention to pursue intermittent therapy. She had a complete response after less than two months of therapy, at which point therapy was held. Short-term toxicities included Grade 2 transaminitis, dysgeusia, and pyrexia. Serial imaging in the 16 months thus far has demonstrated an ongoing response (Figure 1).

## Discussion

Activating BRAF V600E kinase mutation leads to the constitutional activation of signalling in mitogen-activated protein kinases (MAPK) pathway. Vemurafenib and other inhibitors of mutated BRAF have shown remarkable response rates of greater than 90% in BRAF mutant melanoma with improvement in PFS and OS. Unfortunately, resistance is expected and survival beyond two years is uncommon. At the same time, long-term oral dosing can be accompanied by chronic toxicities causing decreased quality of life, including arthralgia, fatigue, diarrhea, and squamous cell carcinoma [1]. Our patient also developed intolerable cutaneous side effects, including blistering of soles and heels, mandating dose reduction and eventually discontinuation of therapy.

Resistance to BRAFi occurs via a variety of mechanisms, including receptor tyrosine kinase (RTK)-mediated activation of alternative survival pathways or reactivation of MAPK pathway via N-RAS upregulation [2]. Secondary mutations in the coding sequence of BRAF, NRAS, KRAS, HRAS, or MEK1 were not demonstrated in resistant tumors. The addition of an MEK inhibitor to a BRAF inhibitor was well tolerated in Phase 1 and 2 trials with improved response rates and PFS [3].

Current data and guidelines support continuous dosing of BRAFi until disease progression. However, laboratory data suggest a role for intermittent therapy. Animal studies have demonstrated that vemurafenib-resistant BRAF V600E melanoma cell lines required vemurafenib for continuous proliferation. Removal of the drug from resistant cells in vitro led to MAPK pathway hyperactivation and resistant cells experience a fitness deficit. Intermittent dosing with vemurafenib was tested in mice and led to better outcomes than continuous dosing [5]. Similarly, when resistance to BRAF and MEK inhibitors was studied, it was found to be associated with acquired epidermal growth factor receptor (EGFR) expression. This EGFR expression was beneficial for proliferation to melanoma cells in the presence of BRAF or MEK inhibitors, and this is reversed upon discontinuation of the drug [6]. These studies suggest that cessation of drug administration may allow for the maintenance of BRAFi efficacy with the ability to retreat after periods off of therapy.

Although intermittent dosing of BRAF inhibitors is conceptually attractive, its widespread acceptance has not occurred due to the lack of clinical trial data. Intermittent therapy can be done as a pre-defined on/off schedule, such as is done in intermittent androgen deprivation therapy in prostate cancer, or on a pro re nata basis where treatment resumption is done on an as needed basis. Recently, six cases of treatment of metastatic melanoma with a variety of schedules of intermittent vemurafenib were reported with improved tolerance and/or stable or progression-free disease [7]. In that series, only three of the six cases reported response beyond 12 months and none for more than two years. In another report of two patients, response of four and five months was reported on rechallenge with BRAFi along with MEKi [8]. However, both these patients had received ipilimumab at some point during their treatment course. To our knowledge, this is the first report of prolonged survival with pro re nata intermittent BRAFi.

## Conclusions

Long-term disease control of four years or more with BRAFi is possible in exceptional circumstances. Preclinical data support that intermittent BRAFi may improve treatment efficacy. We demonstrate that prolonged disease control with limited drug exposure is possible with this approach. Long-term outcomes and validity of intermittent therapy in randomized trials are needed.
